# Acyl-CoA synthetase long-chain 3-mediated fatty acid oxidation is required for TGFβ1-induced epithelial-mesenchymal transition and metastasis of colorectal carcinoma

**DOI:** 10.7150/ijbs.69802

**Published:** 2022-03-14

**Authors:** Jing Quan, Can Cheng, Yue Tan, Nian Jiang, Chaoliang Liao, Weihua Liao, Ya Cao, Xiangjian Luo

**Affiliations:** 1 Key Laboratory of Carcinogenesis and Invasion, Chinese Ministry of Education, Department of Radiology, Xiangya Hospital, Central South University, Changsha, Hunan 410078, PR China; 2 Cancer Research Institute, School of Basic Medicine, Central South University, Changsha, Hunan 410078, PR China; 3 Hengyang Medical College, University of South China, Hengyang 421001, Hunan, PR China; 4 Department of Neurosurgery, Xiangya Hospital, Central South University, Changsha, Hunan 410078, PR China; 5 Hunan Key Laboratory of Oncotarget Gene, Hunan Cancer Hospital and The Affiliated Cancer Hospital of Xiangya School of Medicine, Central South University, Changsha, Hunan 410078, China; 6 Key Laboratory of Biological Nanotechnology of National Health Commission, Central South University, Changsha, Hunan 410078, China; 7 Molecular Imaging Research Center of Central South University, Changsha, Hunan 410078, China; 8 National Clinical Research Center for Geriatric Disorders, Xiangya Hospital, Central South University, Changsha, 410078, China

**Keywords:** Acyl-CoA synthetases 3, Fatty acid β-oxidation, Epithelial-to-mesenchymal transition, Metastasis, Colorectal carcinoma

## Abstract

Cancer cells frequently undergo metabolic reprogramming to support tumorigenicity and malignancy, which is recognized as a hallmark of cancer. In addition to glycolysis and glutaminolysis, alterations in fatty acid (FA) metabolism have received increasing concerns in the past few years. Recently, accumulating evidence has shown that fatty acid β-oxidation (FAO) is abnormally activated in various tumors, which is associated with the machinery of proliferation, stemness, metastasis, and radiochemotherapeutic resistance of cancer cells. Acyl-CoA synthetases 3 (ACSL3) belongs to a family of enzymes responsible for converting free long-chain FAs into fatty acyl-CoA esters, which act as substrates both for lipid synthesis and FAO.

Here, we demonstrate that transforming growth factor beta 1 (TGFβ1) induces the up-regulation of ACSL3 through sterol regulatory element-binding protein 1 (SREBP1) signaling to promote energy metabolic reprogramming in colorectal carcinoma (CRC) cells. ACSL3 mediates the epithelial mesenchymal transition (EMT) and metastasis of CRC cells by activation of FAO pathway to produce ATP and reduced nicotinamide adenine dinucleotide phosphate (NADPH), which sustain redox homeostasis and fuel cancer cells for invasion and distal metastasis. Thus, targeting ACSL3 and FAO metabolic pathways might be exploited for therapeutic gain for CRC and other FAs- addicted cancers.

## Introduction

Cancer cells frequently undergo metabolic reprogramming to support tumorigenicity and malignancy, which is recognized as a hallmark of cancer 1-5. In addition to glycolysis and glutaminolysis, alterations in fatty acid (FA) metabolism have received increasing concerns in the past few years 6-13. Fatty acid β-oxidation (FAO) consists of a cyclic series of reactions resulting in the shortening of fatty acids to generate NADH, FADH_2_, and acetyl-CoA 14, 15. The two formers are coupled to mitochondrial electron transport chain complex (ETC) to produce ATP. The latter enters into the Krebs cycle to generate citrate, which can be exported to the cytoplasm for NADPH production 16. Recently, accumulating evidence has shown that FAO is abnormally activated in a variety of tumors, such as breast cancer, prostate cancer, lung cancer, glioma, and diffuse large B-cell lymphoma (DLBCL), which are associated with the machinery of proliferation, stemness, metastasis and radiochemotherapeutic resistance of cancer cells 17-19. Increased expression of CD36, a fatty acid translocase, and a high FAO rate mediate the resistance of AML cells to cytarabine treatment 20. JAK/STAT3 signaling enhances the stemness and chemotherapy resistance of breast cancer cells by activation of the FAO pathway 21. Our previous studies have demonstrated that the activation of FAO pathway promotes the radiation resistance of nasopharyngeal carcinoma (NPC) cells 19, 22.

Acyl-CoA synthetases long-chain (ACSLs) are a family of enzymes responsible for converting free long-chain FAs into fatty acyl-CoA esters, which is the prerequisite step for FAO. In turn, fatty acyl-CoAs can be transported into the mitochondrial matrix and undergo FAO 23. The five ectopic forms of ACSLs in mammals are ACSL1, 3, 4, 5, and 6, and they prefer fatty acid substrates that generally have a chain length of 12-20 carbon atoms^24^, ^25^. ACSL3 is enriched in lipid droplet and endoplasmic reticulum and plays a critical role both in lipid anabolism and catabolism as well as in fatty acid uptake. In hepatoma cells, ACSL3 was required to synthesize phosphatidylcholine that is essential for very low-density lipoprotein (VLDL) assembly. Depletion of ACSL3 limited VLDL secretion and hepatitis C virus (HCV) infection 26. ACSL3 has also reported as the critical enzyme required for mutant KRAS lung tumorigenesis by facilitating the uptake and β-oxidation of FAs 18. In addition, high ACSL3 expression predicted a poor prognosis and chemotherapy resistance in melanoma 27.

Transforming growth factor β1 (TGFβ1) has been found up-regulated in the microenvironment of various tumors, such as non-small cell lung cancer and breast cancer. Sustained low-dose TGFβ1 exposure can promote epithelial-mesenchymal transition (EMT) of cancer cells 28-30. EMT process is closely associated with the malignant phenotype of cancers, endowing cancer cells the ability to migrate, extravasation, and even spread to distant organs 31. Although glucose metabolism has been extensively investigated in rapidly proliferative cancer cells, the metabolic reprogramming that mediates EMT and metastasis is not fully clarified. In A549 adenocarcinoma NSCLC cells, TGFβ1-induced EMT process is accompanied by reduced lipogenesis and concomitant enhanced mitochondrial respiration^29^. Metastatic triple negative breast cancer cells mainly depend on FAO to maintain high levels of ATP; Inhibition of FAO significantly hampers the invasion and metastasis characteristics of the cells^32^.

Here, we demonstrate that fatty acid uptake and β-oxidation are substantially enhanced during TGFβ1-induced EMT in CRC cells. ACSL3 mediates fatty acid metabolism reprogramming induced by TGFβ1 and promotes the EMT and metastasis of CRC cells. These findings establish the molecular link between energy metabolic reprogramming and invasive phenotype induced by TGFβ1, and indicate that ACSL3 might participate in this process by activating FAO to fuel cell migration and metastases of cancer cells.

## Materials and methods

### Cell culture

The human colorectal carcinoma HCT116 (ATCC CCL-247) and LoVo (ATCC CCL-229) cells were maintained in RPMI-1640 media (Hyclone, UT, USA) containing 10% (v/v) heat-inactivated fetal bovine serum (FBS, Hyclone). The human embryonic kidney HEK293T (ATCC CRL-3216) cells were cultured in Dulbecco's modified eagle medium (Hyclone) supplemented with 10% (v/v) FBS (Hyclone). All cells were maintained in a humidified atmosphere with 5% CO_2_ at 37 °C.

### Reagents and chemicals

We used β-actin from ImmunoWay Biotechnology (Plano, TX, USA) as an internal control. Anti- ACSL3 was derived from Invitrogen (Carlsbad, CA, USA). The antibodies against ZO-1, E-Cadherin, N-Cadherin, Vimentin, and Snail were from Cell Signaling Technologies (Danvers, MA, USA). Anti-SREBP1, anti-rabbit IgG-HRP, and anti-mouse IgG-HRP were obtained from Santa Cruz Biotechnology (CA, USA).

Triacsin C was purchased from abcam (Cambridge, MA, USA), and Etomoxir (ETO) was derived from MedChemExpress (NJ, USA).

### Measurement of glucose consumption and lactate production

We seeded 5 × 10^5^ cells in 6-well plates, cultured them at 37 °C in a 5% CO 2 incubator for 6 hours, and then replaced them with fresh medium for 8 hours. The Automatic Biochemistry Analyzer detected the contents of glucose and lactate (7170A, Hitachi, Japan).

### Flow cytometric analysis of fatty acid uptake

Cells were serum-starved for 3 hours, and BODIPY™ FL_C16_ (4, 4-Difluoro-5,7-Dimethyl-4-Bora-3a,4a-Diaza-s-Indacene-3-Hexadecanoic Acid) (Invitrogen, D3821) bound to bovine serum albumin (BSA) was added. After 30 min incubation at 37°C in the dark incubator, cells were washed with pre-chilled 1X PBS and detached by trypsinization. Cells were finally fixed with 4% paraformaldehyde (PFA). Fatty acid uptake was determined by measuring fluorescence intensity within cells with Becton Dickinson FACS Calibur and analyzed by Cell Quest Pro software (BD Biosciences, Germany).

### Fuel oxidation analysis

The cells' dependency on oxidizing three mitochondrial fuels (glucose, glutamine, long-chain fatty acids) was measured by determining the rate of oxidation of each fuel of cells in the presence or absence of fuel pathway inhibitors. According to the manufacturer's instructions, it was performed using a Mito Fuel Flex Test kit (Agilent).

### UPLC-MS/MS analysis

The UPLC-MS/MS analysis was performed on a Waters Acquity UPLC system (Waters, Milford, MA) coupled to a Triple Quad™ 5500 tandem mass spectrometer (AB Sciex, Framingham, MA), and 5 μL of each sample was injected onto a Thermo HPLC Syncronis AQ C18 column (100mm × 2.1mm, 1.7μm) at a flow rate of 0.4 mL/min. The analysis eluted from the column was ionized in an electrospray ionization source in positive mode (ESI+). The multiple reaction monitoring (MRM) was used to acquire data in optimized MRM transition (precursor > product), declustering potential, and collision energy. Analyst (version 1.5.2, AB Sciex) was used to analyze data. Using the default parameters and assisting manual inspection in ensuring each compound's qualitative and quantitative accuracy, extracting and output chromatographic retention time, and peak area.

### Measurement of FAO

Cells were plated and grown for 8 h in a complete medium. The medium was replaced with a substrate-limited medium for 4 h. After that, cells were replaced with an FAO assay medium and incubated at 37°C for another 45 min. Before starting the assay, 30 µl palmitate-BSA substrate or the control BSA and 10 µl reconstituted MitoXpress® reagent was added to the wells. The oxygen consumption rate (OCR) was detected by an Envision® multimode plate reader (Perkin Elmer) using the MitoXpress® Xtra oxygen consumption assay kit (Luxcel Bioscience), and the slope of the fluorescence curve was set as the OCR value.

### Cellular ATP and NADPH/NADP measurement

The intracellular ATP levels were measured using the CellTiter-Glo 2.0 Assay kit (Promega), and NADPH/NADP levels were assayed using an NADP/NADPH Quantification Colorimetric Kit (Biovision) according to the manufacturer's instructions.

### Measurement of ACSL activity

ACSL activity in total cell lysates was measured by monitoring the incorporation of radiolabeled palmitate into palmitoyl-CoA using previously published protocols 33.

### Oil Red-O staining

Lipid accumulation was measured using Oil Red O (Sigma-Aldrich) staining. Colon cancer cells were plated and cultured in 6-well plates and transfected with plasmids or siRNA plasmids for 48 h. Cells were washed three times with PBS and fixed with 10% paraformaldehyde at room temperature (RT) for 30 min. Then cells were stained with 1.5% Oil Red working solution and incubated for 30 min at RT. The Oil Red working solution was performed according to the manufacturer's instructions. Then 60% isopropanol was added to the cells and incubated for 20 s, followed by three washes with PBS. The stained lipid droplets were observed under a light microscope. The percentage of stained cells to the total cells in the observed field was calculated to quantify the lipid production.

### Three-dimensional (3D) invasion assay

3D cell culture was performed as described previously 34, 35. Matrigel (BD Biosciences) was added to a 24-well plate to cover the bottom of the plate completely. After solidification at 37°C for 45 min, a 200 μl suspension containing 500 cells was dropwise added to the top of the Matrigel and allowed to settle for 1 h. Each well was further supplemented with 200 μl of media containing 10% Matrigel. The invasive degree of spheroids was classified into two types: noninvasive spheroids displaying smooth edges with no or only occasional scattered protrusions; or invasive spheroids with fully scattered protrusions.

### Immunohistochemical analysis

The tissues sections from routine diagnostic biopsy specimens were obtained from Xiang-Ya Hospital of Central South University. Tissues were fixed with 4% formaldehyde, embedded in paraffin, and sectioned. Tumor tissue sections were deparaffinized in a dewaxing agent (solarbio, China) and rehydrated with ethanol-aqueous. Endogenous peroxidase activity was blocked by hydrogen peroxide (3%). The primary antibodies (anti-ACSL3) were applied at 4 ℃ overnight, followed by incubation with anti-rabbit secondary antibody. Chromogen was developed using DAB (Zsgbbio, China) and counterstained with hematoxylin.

### Animal studies

Animal procedures were in accordance with the standards established by the guidelines for the Care and Use of Laboratory Animals of Central South University (Changsha, China). To interrogate whether overexpression of *ACSL3* promotes tumor metastasis *in vivo*, stable HCT116 CON and HCT116 ACSL3 cells (3✕10^6^) were injected into the tail veins of female BALB/c nu/nu mice (5 animals per group, 5 weeks old), respectively. At the end of the experimental period, the mice were euthanized by CO_2_ inhalation, and lung tissue was harvested. The number of metastatic lung nodules was counted, and the metastatic tumors were further confirmed using hematoxylin and eosin (HE) staining as described.

To assess the effect of ACSL3 loss in the metastasis of CRC cells, stable LoVo shCON and LoVo shACSL3 cells were injected into the portal vein of BALB/c nude mice (8 animals per group, 8 weeks old), respectively. After median laparotomy, the hilum of the liver was exposed to access the portal vein. Cells (1✕10^6^) in a 50 μl PBS buffer volume were injected slowly into the portal vein using a 30 G needle. After 4 weeks, all animals were sacrificed. Explanted livers and the laparoscopic metastatic tumor tissues were sliced for further confirmation using HE staining. The ethics committee approved both the present study protocols of Xiangya Medical School of Central South University.

### Statistical analysis

All statistical calculations were performed with the statistical software program SPSS ver.16.0. Differences between various groups were evaluated by a two-tailed Student's t-test and a p-value < 0.05 was considered statistically significant.

## Results

### TGFβ1 induces EMT to promote invasion of CRC cells

**Figure 1:**
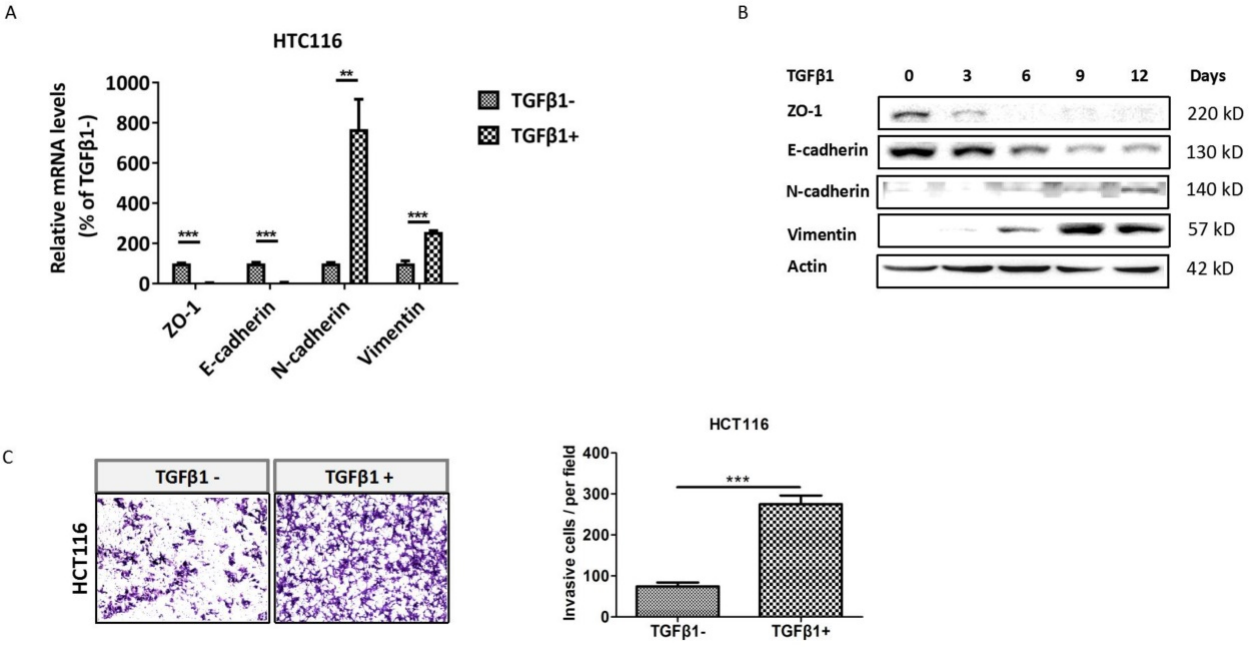
** TGFβ1 induces EMT to promote invasion of CRC cells. HCT116 cells were treated with TGFβ1 (2ng/ml) for the indicated days.** (A) mRNA levels of the EMT functional genes were detected using real-time PCR after HCT116 cells were treated with TGFβ1 for 0 (marked TGFβ1-) or 6 days (marked TGFβ1+). (B) Protein levels of the EMT functional proteins at the indicated time were analyzed by Western blot. (C) The cell invasion ability of HCT116 cells was determined after being treated with TGFβ1 for 0 (marked TGFβ1-) or 6 days (marked TGFβ1+). Data are shown as mean values ± S.D. of independent, triplicate experiments. The asterisks (**, ***) indicate significant differences (*p* < 0.01, *p* < 0.001, respectively).

To identify whether TGFβ1 induces EMT in CRC cells, we detected the expression of EMT markers in HCT116 cells with low dose exposure to TGFβ1 for 0, 3, 6, 9, 12 days, respectively. The results showed that both mRNA and protein levels of ZO-1 and E-cadherin were substantially decreased in HCT116 cells treated with TGFβ1. Conversely, those of N-cadherin and vimentin were markedly enhanced (Figure 1A -1B). Next, cell invasion assay demonstrated that the number of cells invaded through Matrigel dramatically increased after TGFβ1 treatment (Figure 1C). These results indicate that TGFβ1 induces EMT to promote invasion of CRC cells.

### TGFβ1 activates FAO to induce metabolic reprogramming in CRC cells

**Figure 2:**
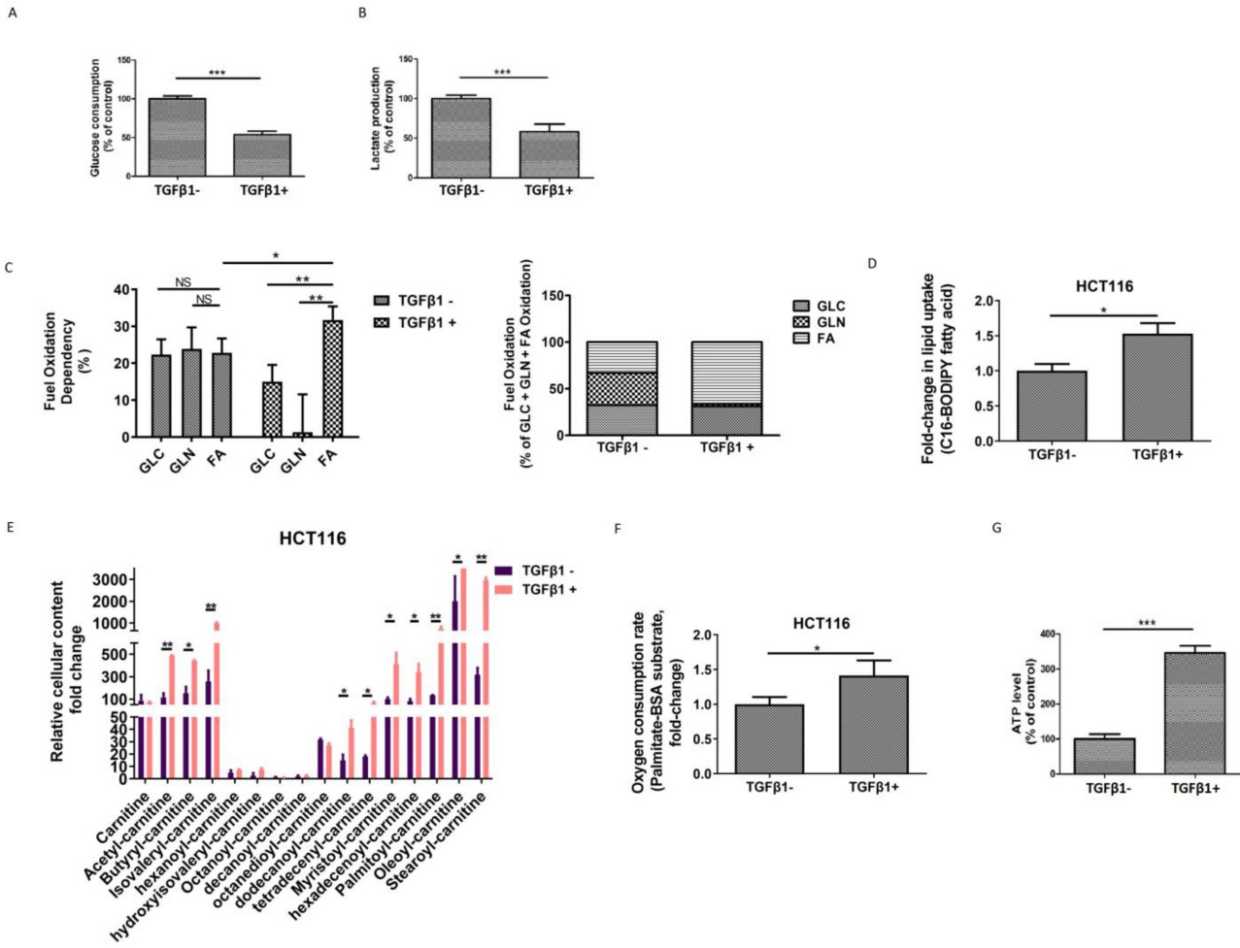
** TGFβ1 activates FAO to induce metabolic reprogramming in CRC cells. HCT116 cells were treated with TGFβ1 for 0 (marked TGFβ1-) or 6 days (marked TGFβ1+).** (A) Glucose consumption and (B) lactate production in HCT116 cells of the designated group. (C) Mito Fuel Flex test showed dependence on the oxidation of glucose (GLC), glutamine (GLN), or fatty acid (FA) in HCT116 cells of the designated group. (D) BODIPY-FA uptake in HCT116 cells treated with TGFβ1 as indicated. (E) UPLC-MS/MS analysis of carnitines in HCT116 cells of the designated group. (F) β-oxidation assay and (G) Intracellular ATP levels in HCT116 cells of the designated groups. Data are shown as mean values ± S.D. of independent, triplicate experiments. The asterisks (**, ***) indicate significant differences (*p* < 0.01, *p* < 0.001, respectively).

To investigate the metabolic changes that occur during TGFβ1-induced EMT, first, we tested the glycolytic levels in HCT116 cells in the absence or presence of TGFβ1. We found that both glucose consumption and lactate production were declined upon TGFβ1 exposure (Figure 2A-2B), which suggests that TGFβ1 treatment inhibits glycolysis in CRC cells. Second, we performed additional assays to further characterize the rewiring metabolic pattern induced by TGFβ1. We adopted a Seahorse mito fuel assay, which determines the dependency of cells on the three different mitochondrial fuels to meet basal energy demands, and illustrated that in the absence of TGFβ1, fuel oxidation relatively evenly depended on glucose, glutamine and fatty acid, whereas TGFβ1 treatment significantly increased overall dependency on oxidation of fatty acids rather than glucose or glutamine in HCT116 cells (Figure 2C). Moreover, Next, we used a fluorescent Bodipy fatty acid probe (C16-BODIPY), a very long-chain fatty acid analog, to measure cellular fatty acid uptake. The data showed that uptake of BODIPY C16 was substantially augmented in HCT116 cells with TGFβ1 treatment (Figure 2D), which is in accordance with TGFβ1-induced increased dependency on the FAO pathway.

Carnitines, as acyl-CoA carriers, are responsible for transferring FAs into the mitochondrial matrix. The production of acyl-carnitines facilitates the activation of FAO. Using UPLC-MS/MS analysis, we found that several acylcarnitines, including acetyl-carnitine, butyryl-carnitine, isovaleryl-carnitine, dodecanoyl-carnitine, tetradecenyl-carnitine, myristoyl-carnitine, hexadecenoyl-carnitine, palmitoyl-carnitine, oleoyl-carnitine, and stearoyl-carnitine were dramatically up-regulated induced by TGFβ1 (Figure 2E). To further confirm whether the FAO pathway is activated, we measured the palmitate (PA)-based oxygen consumption rate (OCR) in the absence or presence of TGFβ1. After TGFβ1 treatment, significant increases in OCR and intracellular ATP levels were observed (Figure 2F-2G). Together, these findings support that TGFβ1 treatment up-regulates FAs uptake and induces the activation of the FAO pathway, resulting in metabolic rewiring in CRC cells.

### TGFβ1 up-regulates ACSL3 to promote the EMT and invasion of CRC cells

**Figure 3:**
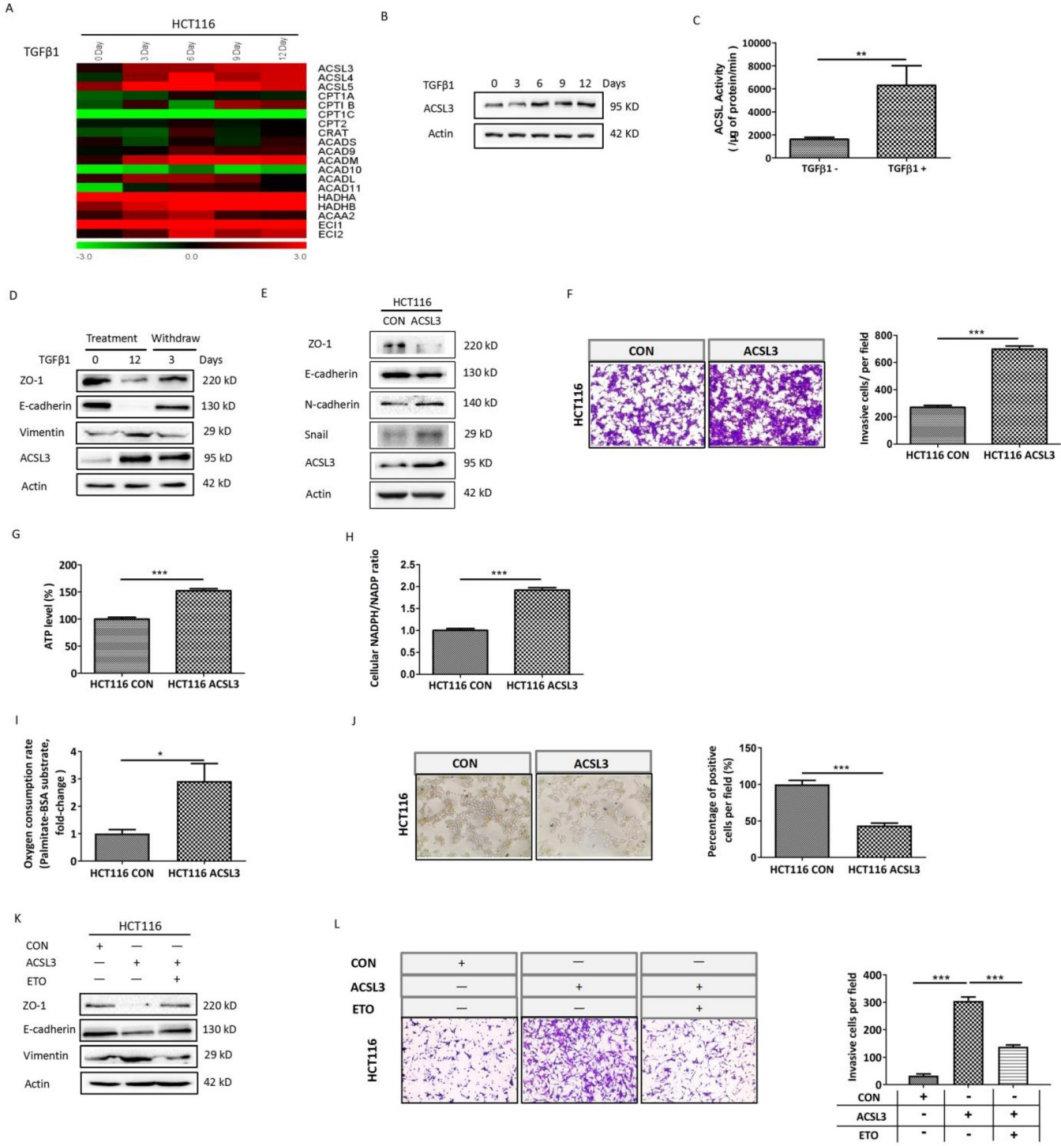
** TGFβ1 up-regulates ACSL3 to promote the EMT and invasion of CRC cells.** (A) The heatmaps illustrated the changes in expression of FAO genes in HCT116 cells treated with TGFβ1 as indicated. The expression level shown is representative of log2 values of each replicate. Red represents higher expression and green for lower expression relative to the mean expression level within the group. (B) ACSL3 protein expression in HCT116 cells treated with TGFβ1 as indicated. (C) HCT116 cells were treated with TGFβ1 for 0 (marked TGFβ1-) or 6 days (marked TGFβ1+), and ACSL enzymatic activity was measured in each designated group. (D) HCT116 cells were treated with TGFβ1 for 0, 12 days, respectively. After that, TGFβ1-treated HCT116 cells were cultured in a normal growth medium without TGFβ1 for 3 days. Protein levels of the EMT functional proteins (ZO-1, E-cadherin, Vimentin) and ACSL3 were analyzed by western blot. (E) Protein levels of the EMT functional proteins and ACSL3 in HCT116 CON and HCT116 ACSL3 cells. (F) The cell invasion ability increased in ACSL3-transfected HCT116 cells compared with vector control cells. (G) Intracellular ATP levels, (H) NADPH/NADP ratio, (I) β-oxidation assay, and (J) Representative images of Oil Red O staining in HCT116 CON and HCT116 ACSL3 cells. (K) Protein levels of the EMT functional proteins (ZO-1, E-cadherin, Vimentin) in HCT116 CON and HCT116 ACSL3 cells with or without ETO (100 μM) treatment as indicated. (L) The cell invasion ability was detected in HCT116 CON and HCT116 ACSL3 cells with or without ETO (100 μM) treatment as indicated. Data are shown as mean values S.D. of independent, triplicate experiments. The asterisks (*, **, ***) indicate significant differences (*p* < 0.05, *p* < 0.01, *p* < 0.001, respectively).

To gain insight into the mechanism of TGFβ1-induced FAO activation, the transcriptional profile of genes involved in the FAO pathway was screened in HCT116 cells with TGFβ1 exposure for 0, 3, 6, 9, 12 days, respectively. The data showed that *ACSL3* mRNA was gradually up-regulated with the exposure time prolonged (Figure 3A). Consistent with this, the protein levels of ACSL3 were also induced by TGFβ1 in a time-dependent manner (Figure 3B). Furthermore, we confirmed that HCT116 cells exposure to TGFβ1 also displayed a substantial enhancement of ACSL enzymatic activity compared to the control (Figure 3C).

TGFβ1-induced EMT is a reversible process. We observed up-regulation of ACLS3 and decreases in ZO-1 and E-cadherin with concomitant increases in vimentin during TGFβ1 treatment, whereas these responses were reversed after TGFβ1 withdrawal. These results further support that ACSL3 expression is TGFβ1-inducible (Figure 3D). Moreover, overexpression of *ACLS3* substantially down-regulated ZO-1 and E-cadherin while up-regulated N-cadherin and snail (Figure 3E). Using cell invasion assay, we observed that the number of cells invaded significantly increased in the ACLS3-transfected HCT116 cells compared to the control (Figure 3F).

To determine whether ACSL3 activates FAO to promote EMT and invasion in CRC cells, we tested the metabolic change in ACLS3-transfected HCT116 cells compared to the control. Both ATP content and NADPH/NADP ratio were markedly augmented in ACLS3-transfected cells (Figure 3G-3H). Moreover, overexpression of *ACLS3* significantly increased PA-based OCR approximately by 3-fold (Figure 3I). In addition, Oil Red-O staining illustrated a significant decrease of lipid droplet accumulation in ACLS3-transfected cells compared to the control (Figure 3J), which suggests that ACSL3 attenuates anabolism and promotes catabolism of FAs. These data support that *ACLS3* overexpression promotes the activation of the FAO pathway. We further used a specific inhibitor of FAO, Etomoxir (ETO), to investigate whether FAO mediates the ACSL3-induced enhancement of cell invasion. We found that ETO treatment effectively reversed EMT and cell invasion induced by overexpression of ACSL3 (Figure 3K-3L). These findings support that TGFβ1 up-regulates ACSL3 protein expression and its enzymatic activity to boost FAO, resulting in the enhancement of aggressive capability of CRC cells.

### ACSL3 knockdown impairs the EMT and invasion of CRC cells

**Figure 4:**
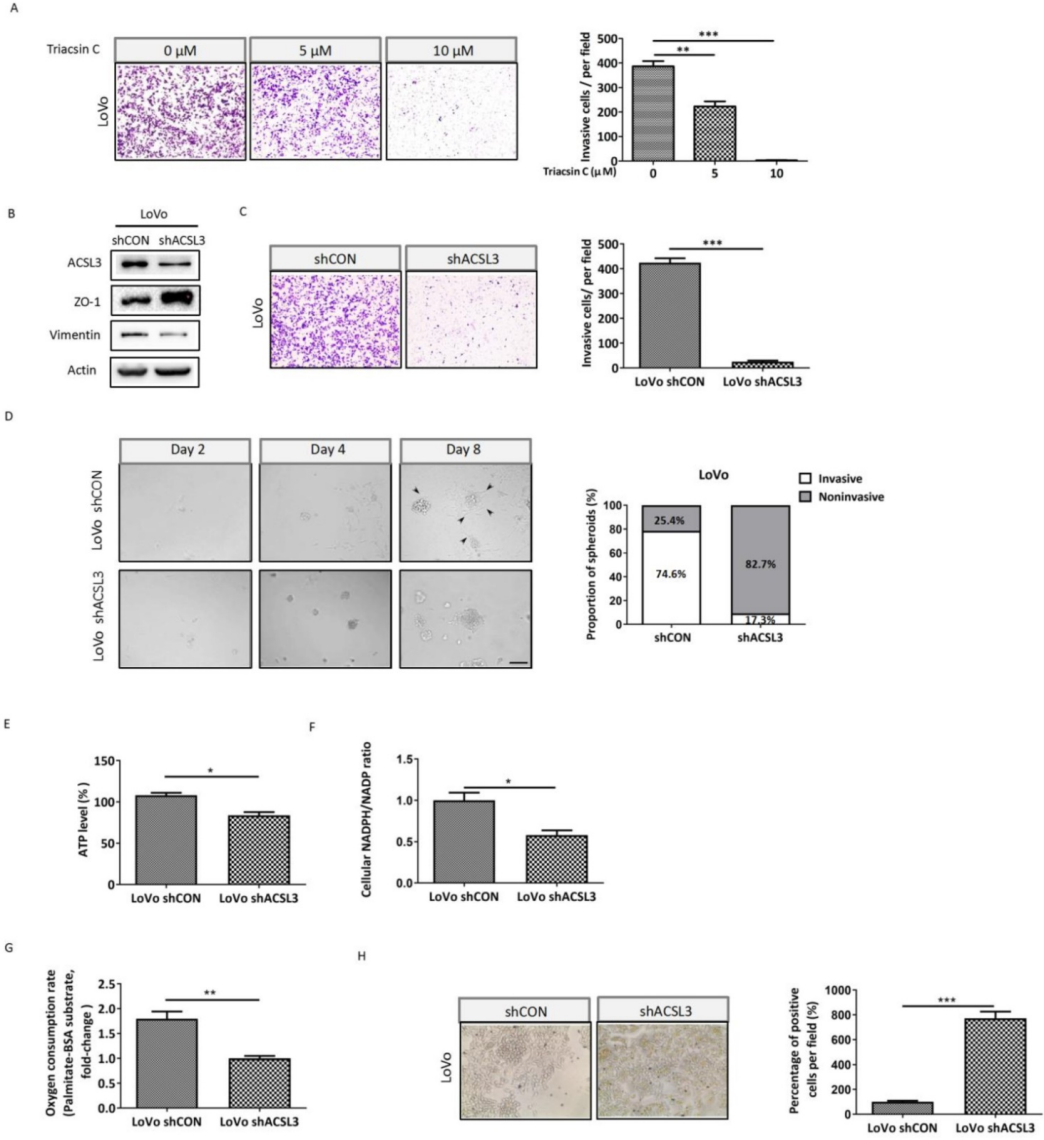
** ACSL3 knockdown attenuates FAO to impair the EMT and invasion of CRC cells.** (A) LoVo cells were treated with Triacsin C (0, 5, 10 μM) for 48 h, and the cell invasion abilities were detected. (B) Protein levels of the EMT functional proteins and ACSL3 in LoVo shCON and LoVo shACSL3 cells. (C) The cell invasion ability was detected in LoVo shCON and LoVo shACSL3 cells. (D) Representative images of spheroids formed by LoVo cells in Matrigel (left). The invasive spheroids were featured by scattered protrusions formed on the spheroid surface, as indicated by black arrows. Scale bar, 50 μm. Proportion of two spheroid types in LoVo shCON and LoVo shACSL3 cells (right) (*p* <0.01). (E) Intracellular ATP levels, (F) NADPH/NADP ratio, (G) β-oxidation assay, and (H) Representative images of Oil Red O staining in LoVo shCON and LoVo shACSL3 cells. Data are shown as mean values S.D. of independent, triplicate experiments. The asterisks (*, **, ***) indicate significant differences (*p*< 0.05,* p<* 0.01, *p*< 0.001, respectively).

To further interrogate the significance of ACSL3 in mediating EMT and invasion of CRC cells, we performed a cell invasion assay using Triacsin C, an inhibitor of ACSL. The results showed that the number of cells invaded was dose-dependently reduced upon Triacsin C treatment in metastatic CRC LoVo cells (Figure 4A). In accordance with this, knockdown of *ACSL3* using small hairpin RNA (shRNA) also reversed EMT and dramatically weakened the invasive capability of LoVo cells (Figure 4B-4C). To mimic the extracellular microenvironment and reproduce the process of cell adhesion and invasion, we used a 3D Matrigel cell culture model. We found that LoVo cells formed invasive spheroids with fully scattered protrusions. However, the knockdown of *ACSL3* attenuated the invasive ability of the spheroids, which displayed relatively smooth edges with fewer protrusions (Figure 4D). These findings demonstrated that ACSL3 mediated the invasion of CRC cells both in monolayer and 3D cell-culture systems.

Next, we explored the metabolic change after knockdown of *ACSL3* and observed that both ATP production and NADPH/NADP ratio were significantly suppressed in LoVo-shACLS3 cells in comparison with the control (Figure 4E-4F). Moreover, the depletion of *ACLS3* substantially decreased PA-based OCR by 1.8-fold (Figure 4G). The results of Oil Red-O staining also supported that *ACSL3* knockdown enhanced FA accumulation (Figure 4H). Taken together, these data further support the notion that ACSL3 induces FAs metabolic reprogramming might contribute to the EMT and invasion of CRC cells.

### SREBP1 mediates the up-regulation of ACSL3 induced by TGFβ1

**Figure 5:**
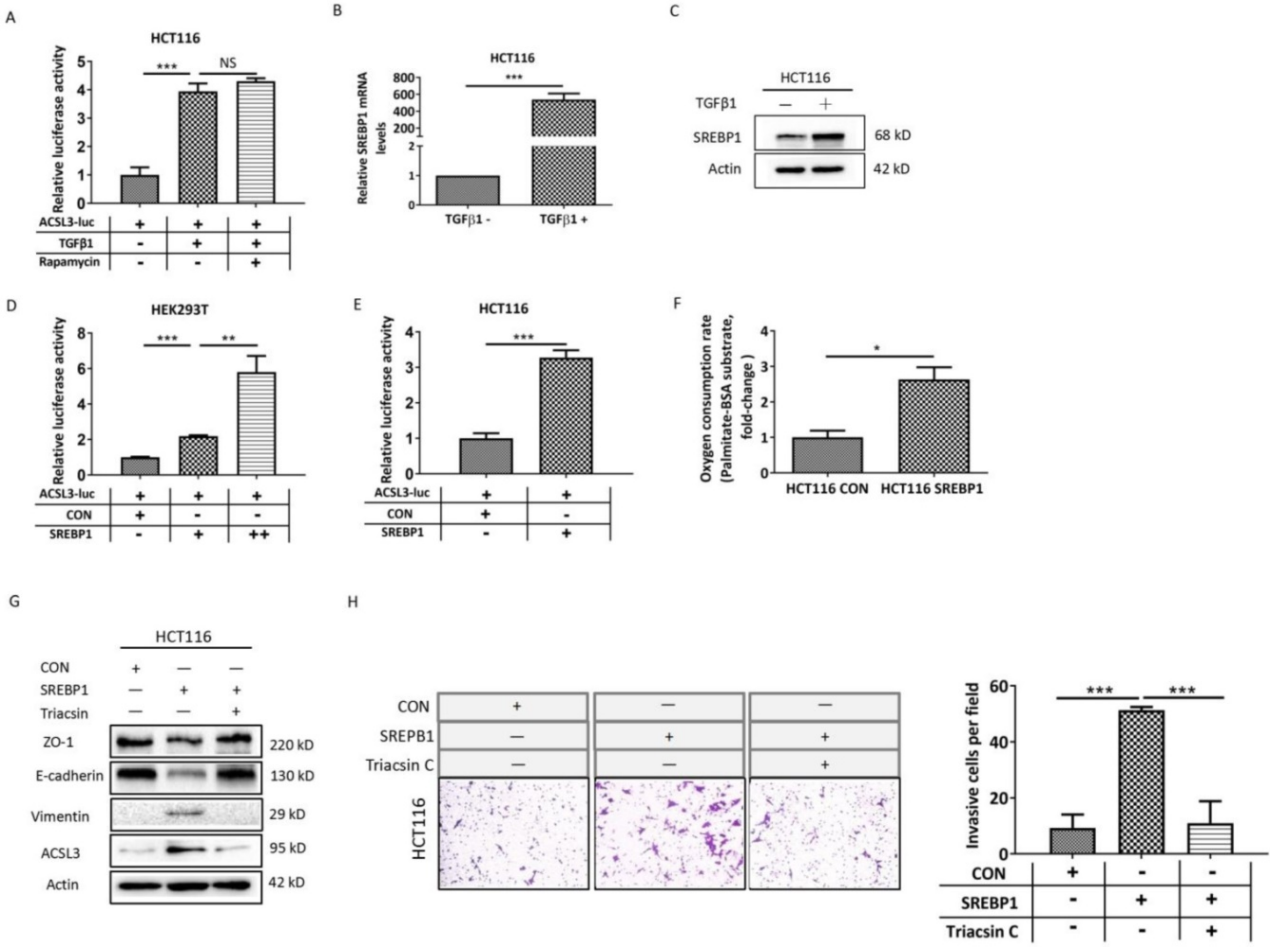
** SREBP1 mediates the up-regulation of ACSL3 induced by TGFβ1.** (A) HCT116 cells were exposed to TGFβ1 (0, 2ng/ml) for 6 days, followed by treatment with rapamycin (0, 1μM) for 48h in each indicated group. Firefly luciferase activity reflecting ACSL3 promoter activity was measured and normalized to renilla luciferase activity. (B) The mRNA and (C) protein levels of SREBP1 in HCT116 cells treated with TGFβ1 for 0 (TGFβ1-) or 6 days (TGFβ1+). After co-transfection with ACSL3-Luc and SREBP1-expression construct into (D) HEK293T and (E) HCT116 cells, respectively, firefly luciferase activity reflecting ACSL3 promoter activity was measured and normalized to renilla luciferase activity. (F) β-oxidation assay in the control and SREBP1-overexpressing HCT116 cells. (G) Protein levels of the EMT functional proteins (ZO-1, E-cadherin, Vimentin) and ACSL3 and (H) the cell invasion ability in HCT116 CON and HCT116 SREBP1 cells. Data are shown as mean values S.D. of independent, triplicate experiments. The asterisks (*, **, ***) indicate significant differences (*p*< 0.05, *p*< 0.01, *p*< 0.001, respectively).

In order to investigate the mechanism of ACSL3 activation induced by TGFβ1, we performed transactivation assays in HCT116 cells and observed that TGFβ1 increases approximately 4-fold the activity of an ACSL3 promoter-reporter (ACSL3-Luc; Figure 5A) compared to the control. Next, the transcription factor and signaling molecular profile associated with EMT was screened out using PCR array assay and was further confirmed by Western blot analysis. We found that the mTORC1 pathway was apparently up-regulated by TGFβ1 as the exposure time extended (Supplementary Figure 1A-1B). Whereas treatment with rapamycin, a well-known inhibitor of mTORC1, did not reverse the ACSL3-Luc activity induced by TGFβ1 (Figure 5A). This result suggests that the mTORC1 pathway might not be involved in TGFβ1-induced ACSL3 expression. Considering that the ACSL3 promoter contains sterol regulatory element-binding protein (SREBP)-binding sites, we further tested whether SREBPs mediated the transcriptional regulation of ACSL3 by TGFβ1. We observed that SREBP1 expression was dramatically increased after TGFβ1 treatment compared to SREBP2 or the control (Figure 5B-5C, Supplementary Figure 1B). The transactivation assay confirmed that SREBP1 overexpression up-regulated the activity of ACSL3-Luc in a dose-dependent manner (Figure 5D). Similar result was also found in HCT116 cells (Figure 5E). Moreover, SREBP1-specific site mutagenesis reversed the augmented ACSL3-Luc activity induced by SREBP1 (Supplementary Figure 3). We observed that SREBP1 overexpression markedly increased PA-based OCR, suggesting an enhanced FAO activity (Figure 5F). In addition, cell invasion assay showed that overexpression of SREBP1 promoted EMT and the invasion of HCT116 cells, which was attenuated by Triacsin C treatment (Figure 5G-5H). These data indicate that TGFβ1 up-regulates SREBP1 to transactivate ACSL3 resulting in FAO activation, which enhances the invasion of CRC cells.

### ACSL3 promotes tumor metastasis *in vivo*

**Figure 6:**
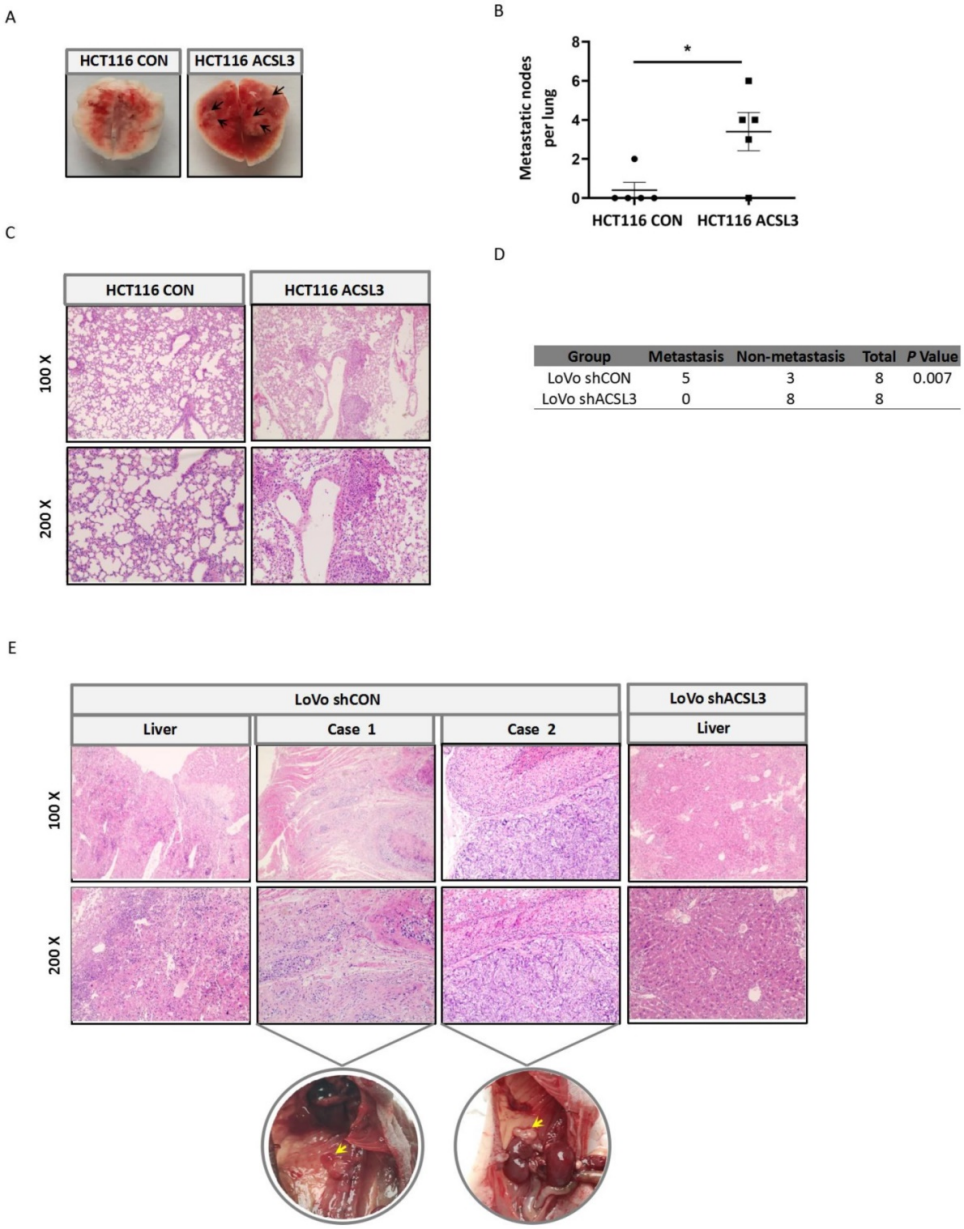
** ACSL3 promotes tumor metastasis *in vivo*. Stable ACSL3-overexpression (ACSL3) or control (CON) HCT116 cells were injected via tail veins of BALB/c nude mice.** (A) The representative lungs from each group showing metastatic tumors (black arrows). (B) The scatter diagram shows the average number of lung surface metastases per mouse. (C) The representative H&E staining of lung tissue sections from each group. (D) Stable shCON or shACSL3 cells were injected into the portal vein of BALB/c nude mice. The metastatic rates of the control and shACSL3 groups were analyzed by Chi-square test (*p* = 0.007). (E) The representative H&E staining of the liver, anadesma near the spine (case 1), and adrenal gland (case 2) tissue sections from each group. The corresponding circled region is representative pictures of metastatic tumor tissues as indicated by yellow arrows.

To interrogate the role of ACSL3 in tumor metastasis *in vivo*, first, stable HCT116 CON and HCT116 ACSL3 cells were injected into the tail veins of BALB/c nude mice, respectively. Overexpression of *ACSL3* resulted in a significant increase of lung metastasis compared to the control (Figure 6A-6B). Hematoxylin-eosin (HE) staining further confirmed this conclusion (Figure 6C). Next, we assessed the effect of ACSL3 loss in the metastasis of CRC cells. Stable shCON and shACSL3 cells were injected into the portal vein of BALB/c nude mice, respectively. Mice in the shCON group showed scattered laparoscopic metastasis, such as anadesma near the spine, adrenal gland, and peritoneum. In contrast, no metastatic colonization was found with mice in the shACSL3 group (Figure 6D). In addition, HE staining demonstrated serious liver inflammation with plenty of lymphocytes infiltration in animals of the shCON group, but not in that of the shACSL3 group (Figure 6E). Therefore, we conclude that ACSL3 exerts a significant role in enhancing CRC metastasis *in vivo*.

**Figure 7:**
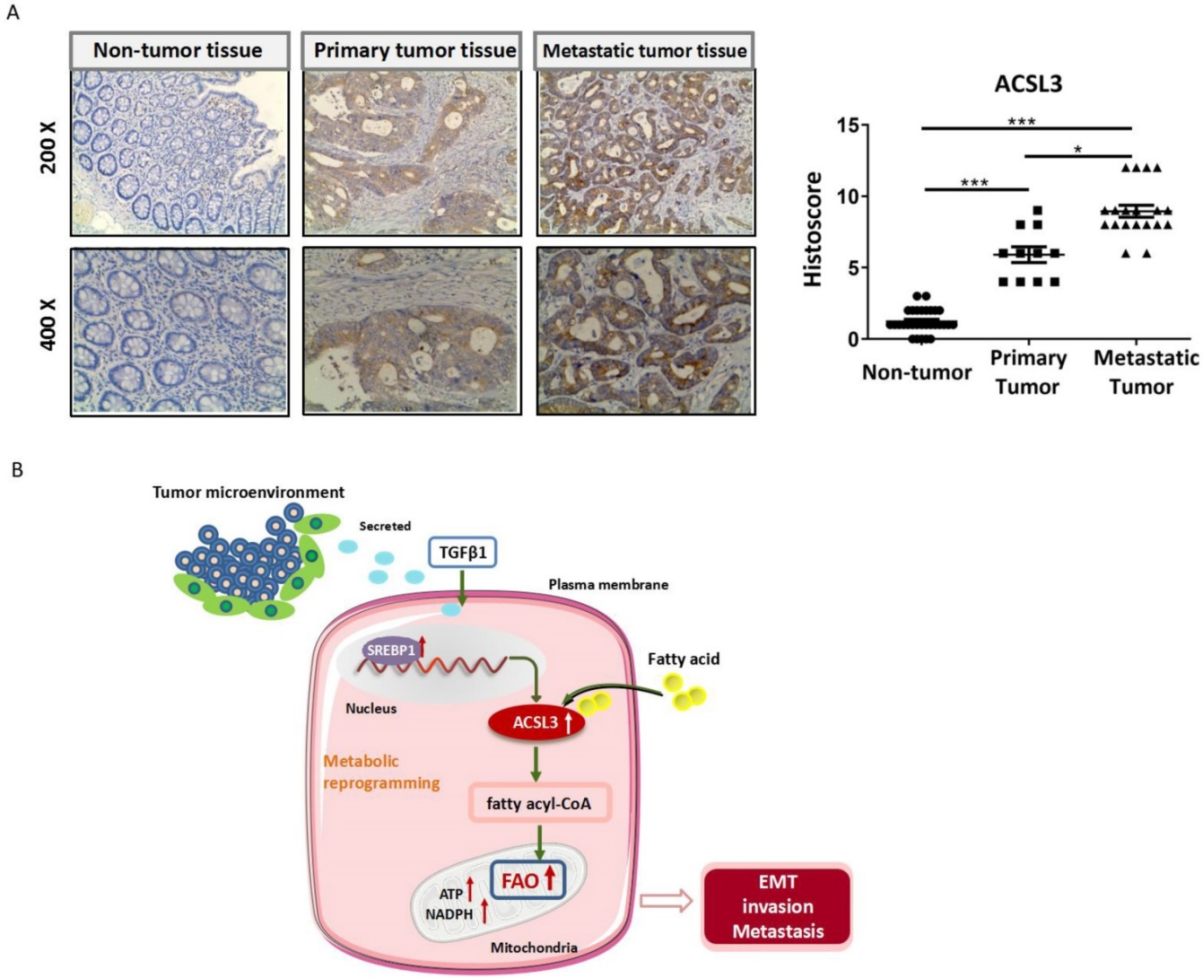
** ACSL3 expression positively correlates with the malignancy in CRC patients.** (A) The representative pictures of ACSL3 staining in primary and metastatic CRC tumor tissues and paired non-tumor tissue. The scatter diagram shows Histoscore for ACSL3 staining in the designated samples. (B) Schematic representation of the mechanism that ACSL3 mediated fatty acid oxidation is required for TGFβ1-induced EMT and tumor metastasis. TGFβ1 induces ACSL3 expression by up-regulating SREBP1, which activates FAO to promote energy metabolic reprogramming, thus facilitating CRC cells to undergo EMT, invasion, and distant metastasis.

To assess whether ACSL3 has any significance in CRC patients, we detected the protein level of ACSL3 in 30 pairs of tumor and non-tumor specimens by immunohistochemistry (IHC). Compared with the corresponding non-tumor colorectal tissues, a significant up-regulation of ACSL3 expression was detected in the CRC specimens (Figure 7A-7B). A scoring scale was applied to quantify ACSL3 staining, which combines the staining intensity with the percentage of positive cells (Histoscore). We adopted the median of H-scores as a threshold and considered an H-score of less than and above it having a low or high staining intensity, respectively. Pearson correlation analysis revealed that ACSL3 expression was positively correlated with distal metastasis of CRC (*p*<0.05, Table 1). However, there was no significant difference in ACSL3 expression in association with age, gender, or tumor differentiation (Table 1). Overall, these results support that high ACSL3 expression contributes to the metastatic progression in CRC patients.

## Discussion

During the process of invasion and distal spread, reprogramming of energy metabolism is needed for cancer cells to overcome the energy crisis, sustaining survival and forming metastasis. In addition to glucose and glutamine, fatty acids also represent an extremely essential energy source. In fact, FAO represents a highly efficient manner for energy provision, in that FAs per dry mass produce twice as much ATP as carbohydrates. Fatty acids mobilized from lipid storage can be catabolized by β-oxidation to provide energy and NADPH, supporting the malignant phenotype of tumors.

In the present study, we have confirmed that TGFβ1 treatment induces EMT and enhances the aggressiveness of CRC cells. Both glucose consumption and lactate production are decreased upon TGFβ1 exposure, which indicates that TGFβ1 treatment inhibits glycolysis. Furthermore, we demonstrated that in the absence of TGFβ1, fuel oxidation relatively evenly depends on the glucose, glutamine, and fatty acid, whereas TGFβ1 treatment substantially increases fuel oxidation dependency on FAs rather than glucose or glutamine. Moreover, TGFβ1 exposure increases FAs uptake. Correspondingly, the content of acyl-carnitines and FA β-oxidation rate are markedly up-regulated with TGFβ1 treatment. Taken together, these findings support the notion that in CRC cells, TGFβ1 treatment triggers metabolic reprogramming, and cells switch to undergo FAO, but not glycolysis or glutaminolysis, to meet the energy need during the process of EMT and invasion. Actually, similar metabolic shift has also been observed in nasopharyngeal carcinoma CNE1 cells during TGFβ1-induced EMT (data not shown). In non-small cell lung carcinoma cells, a metabolic transition that blocks fatty acid synthesis and favors energy production has been found a crucial component of TGFβ1-induced EMT and metastasis 29. FA anabolism and catabolism are opposite metabolic processes, and this observation also provides evidence for lipid metabolic reprogramming in the EMT of cancer cells.

Through screening the transcription profile of lipid metabolism-related genes, we found that *ACSL3* is among the most significantly up-regulated genes during the process of EMT induced by TGFβ1. ACSL3 is placed at a critical intersection of lipid anabolic and catabolic pathways, which is responsible for activating long-chain FAs to provide substrates for both lipid synthesis and β-oxidation 7, 36. ACSL3 is closely associated with tumorigenesis and malignant progression in a variety of cancers 37-40. In our work, we illustrate that TGFβ1 treatment increases both the protein expression and enzymatic activity of ACSL3. Overexpression of ACSL3 enhances FAO as well as the production of ATP and NADPH to promote the EMT and invasion of CRC cells, and vice versa. Consistently, lipid droplet formation is negatively correlated with ACSL3 expression. It further supports that in this scenario, ACSL3 mediates the catabolism of FAs to fuel the invasive progression of CRC cells. ETO treatment reverses the EMT and invasion of CRC cells induced by ACSL3, which suggests that FAO activation is essential for ACSL3 to exert promoting invasion function. Moreover, we have confirmed that ACSL3 is required for tumor metastasis *in vivo*. ACSL3 is up-regulated in CRC tissues in comparison to the corresponding non-tumor control. ACSL3 expression is positively correlated with CRC metastasis. These data indicate that ACSL3 is required for the EMT, invasion, and metastasis of CRC cells by activating the FAO pathway both* in vitro* and *in vivo*.

The results of our transactivation assays show that TGFβ1 exposure up-regulates the transcriptional activity of *ACSL3*. mTORC1 signaling pathway has been reported to mediate the up-regulation of ACSL3 expression in mutant KRAS lung cancer cells 18. Although the mTORC1 pathway is activated upon TGFβ1 exposure, treatment with mTORC1 inhibitor rapamycin shows no change in the transcriptional activity of *ACSL3* promoter, which suggests that mTORC1 signaling might not participate in the transcriptional activation of ACSL3 induced by TGFβ1. Transcription factors SREBPs extensively regulate the expression of genes involved in lipid metabolism. We further illustrate that SREBP1 is substantially up-regulated by TGFβ1 and boosts *ACSL3* transcription in a dose-dependent manner.

Overall, our report provides the important demonstration that TGFβ1 induces the up-regulation of ACSL3 through SREBP1 signaling to promote energy metabolic reprogramming in CRC cells. ACSL3 mediates the EMT and metastasis of CRC cells by activation of the FAO pathway to produce ATP and NADPH, which sustain redox homeostasis and fuel cancer cells for invasion and distal metastasis. Thus, targeting ACSL3 and FAO metabolic pathways might be exploited for therapeutic gain for CRC and other FAs-dependent cancers.

## Supplementary Material

Supplementary methods, figures and table.

## Figures and Tables

**Table 1:** Correlation analysis of ACSL3 up-regulation with clinicopathologic characteristics of CRC patients.

Clinicopathologic characteristics	ACSL3 up-regulation	*P* value
**Age(years)**		0.873
<60	10/14 (71.4%)	
>=60	11/16 (68.7%)	
**Gender**		1
Male	14/20 (70%)	
Female	7/10 (70%)	
**Tumor differentiation**		0.107
Well	1/4 (25%)	
Moderate	14/18 (77.8%)	
Poor	6/8 (75%)	
**Metastasis**		0.019
No	3/8 (37.5%)	
Yes	18/22 (81.8%)	
