# Supplementary material for: Acyl-CoA synthetase long-chain 3-mediated fatty acid oxidation is required for TGFβ1-induced epithelial-mesenchymal transition and metastasis of colorectal carcinoma
Source: Int J Biol Sci. 2022 Mar 14;18(6):2484–96. doi: 10.7150/ijbs.69802 (PMC8990460; doi:10.7150/ijbs.69802)
Supplement: Supplementary file 1 — Supplementary methods, figures and table. [file ijbsv18p2484s1.pdf]

Supplemental Materials and Methods

RNA extraction and quantitative real-time polymerase chain reaction (q-PCR)

Total RNA was extracted using the Nucleozol reagent (Macherey-nagel GmbH & Co, Düren, Germany) according to the protocol established by the manufacturer. Reverse transcriptional PCR was performed using the RevertAid First Strand cDNA Synthesis kit. The qPCR analysis was conducted in a 7500 Real Time PCR System (Applied BioSystems) using the SYBR Green PCR Supermix (Thermo Fisher Scientific). The PCR reaction conditions were 10 s at 95 °C followed by 40 cycles of 15 s at 95 °C and 60 s at 60°C.

Primers and shRNA sequence:

ACSL3-F	5′-CATCGCCATCTTCTGTGAGA-3′
ACSL3-R	5′-GGTGGCTTTCCATCAACAGT-3′
CDH1-F	5′-GCCCCATCAGGCCTCCGTTT-3′
CDH1-R	5′-ACCTTGCCTTCTTTGTCTTTGTTGGA-3′
TJP1-F	5′-CACGCAGTTACGAGCAAG-3′
TJP1-R	5′-TGAAGGTATCAGCGGAGG-3′
CDH2-F	5′-TGGACCATCACTCGGCTTA-3′
CDH2-R	5′-ACACTGGCAAACCTTCACG-3′
VIM-F	5′-CCTGAACCTGAGGGAAACTAA-3′
VIM -R	5′-GCAGAAAGGCACTTGAAAGC-3′
HADHA-F	5′-CTGCCCAAATGGTGGGTGT-3′
HADHA-R	5′-GGAGGTTTAGTCCTGGTCCC-3′
HADHB-F	5′-CTGTCCAGACCAAAACGAAGAA-3′
HADHB-R	5′-CGATGCAACAAACCCGTAAGC-3′
ACSL4-F	5′-ACTGGCCGACCTAAGGGAG-3′
ACSL4-R	5′-GCCAAAGGCAAGTAGCCAATA-3′
ACSL5-F	5′-TGGCTATCTTACAAACAGGTGTC-3′
ACSL5-R	5′-TCCACTCTGGCCTATTCTGAG-3′
CPT1A-F	5′-ATCAATCGGACTCTGGAAACGG-3′
CPT1A-R	5′-TCAGGGAGTAGCGCATGGT-3′
CPT1B-F	5′-CCTGCTACATGGCAACTGCTA-3′
CPT1B-R	5′-AGAGGTGCCCAATGATGGGA-3′
CPT1C-F	5′-GGGCCGCTTTCTTTGTGTC-3′
CPT1C-R	5′-AGAAGACGATTAGGGTGAAGGAT-3′
CPT2-F	5′-CATACAAGCTACATTTCGGGACC-3′
CPT2-R	5′-AGCCCGGAGTGTCTTCAGAA-3′
ACAD9-F	5′-CTCAAGACTAGGGGAGATCATCA-3′
ACAD9-R	5′-ACGCCAGTTTAGGCAAGTATTT-3′
ACAD10-F	5′-CGAGTGGCAAAGCAGTTCC-3′
ACAD10-R	5′-CCATGCAGGACTCCACAATCA-3′
ACAD11-F	5′-TTGGATTCCCCGTTCCCAAG-3′
ACAD11-R	5′-AAATCACGGAAGATTCGACCC-3′
CRAT-F	5′-GTGGCTCAAGACCGCCTAC-3′
CRAT-R	5′-GCAGCAAATCGGAGCTGAC-3′
ECL1-F	5′-CTGCGGTTGTACCAGTCCAA-3′
ECL1-R	5′-GATGCGGTAGTCACAGGTCA-3′
ECL2-F	5′-ATGGGACGCATGGAATGCC-3′
ECL2-R	5′-TTCAAACCCAGTTGATTTCCTGT-3′
ACADM-F	5′-ACAGGGGTTCAGACTGCTATT-3′
ACADM-R	5′-TCCTCCGTTGGTTATCCACAT-3′
ACADS-F	5′-AGGGCGACTCATGGGTTCT-3′
ACADS-R	5′-GGGATGCGACAGTCCTCAAAG-3′
ACADVL-F	5′-TCAGAGCATCGGTTTCAAAGG-3′
ACADVL-R	5′-AGGGCTCGGTTAGACAGAAAG-3′

ACAA2-F	5′-AGACAATGCAGGTAGACGAGC-3′
ACAA2-R	5′-ACCCATGATAGAGGGATCACATC-3′
ACSL3-shRNA	GGATCCACAGGACTTCCAAAG

Western blot

Cells were harvested and washed twice with ice-cold PBS, and then lysed in whole-cell extract buffer (25mM Tris–HCl, pH 7.4, 150mM NaCl, 1%NP40, 1mM EDTA, 5% v/v glycerol). Equal amounts of the total proteins from cell preparations and PageRuler™ molecular weight markers (Fermentas life sciences) were resolved by SDS–polyacrylamide gel electrophoresis and electrotransferred to a PVDF membrane. The membranes were blocked and then incubated with specific primary antibodies according to the manufacturer's recommendations. The primary antibody complexes were then stained with horseradish peroxidase conjugated secondary antibody and developed with the enhanced chemiluminescence detection kit (ECL;Pierce). Visualization was performed using the ChemiDoc XRS system with Image Lab software (Bio-Rad, CA, USA).

Cell invasion assay

The inside of the 8.0 µm-pore cell culture insert was coated with 0.75 mg/ml matrigel (BD Biosciences) at 37 °C for 1 h. 5.0×10^4 cells in serum-free media were inoculated inside each chamber. The cells were invaded with 10% FBS for 72 h. The chamber filter was fixed in 4% paraformaldehyde for 30 min and stained with 0.1% crystal violet. The number of penetrated cells was counted under microscope and three independent assays were repeated.

Luciferase reporter assay

Cells were transfected with 500 ng of reporter constructs (ACSL3-luc plasmid) and 10 ng of the pRL-TK plasmid (Promega). At 48 h after transfection, the cells were disrupted and firefly luciferase activity was measured by using a dual luciferase reporter gene assay kit (Promega) according to manufacturer's instructions.

Supplementary figure legends

Supplementary Figure 1 (A) The heatmap illustrated the changes in expression of EMT-associated transcriptional factor genes in HCT116 cells treated with TGFβ1 as indicated. Expression level shown is representative of log2 values of each replicate. Red represents for higher expression and green for lower expression relative to the mean expression level within the group. (B) HCT116 cells were treated with TGFβ1 (2ng/ml) for the indicated days. Protein levels of the EMT-associated transcriptional factors and signaling proteins at the indicated time were analyzed by Western blot.

Supplementary Figure 2 SREBP2 mRNAs were determined in HCT116 cells treated with TGFβ1 for 0 (TGFβ1-) or 6 days (TGFβ1+).

Supplementary Figure 3

(A) The schematic of the putative SREBP1-binding site for ACSL3 transcription. The continuous point mutation sequence is indicated in red. After co-transfection ACSL3-Luc or ACSL3-Luc Mut with SREBP1-expression construct into (B) HEK293T and (C) HCT116 cells, respectively, firefly luciferase activity reflecting ACSL3 promoter activity was measured and normalized to renilla luciferase activity.

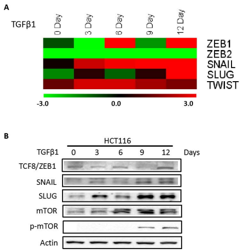
A
TGFβ1
0 Day
3 Day
6 Day
9 Day
12 Day
ZEB1
ZEB2
SNAIL
SLUG
TWIST
-3.0
0.0
3.0
B
HCT116
TGFβ1
0
3
6
9
12
Days
TCF8/ZEB1
SNAIL
SLUG
mTOR
p-mTOR
Actin

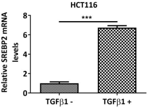
HCT116
Relative SREBP2 mRNA levels
***
0
2
4
6
8
TGFβ1 -
TGFβ1 +

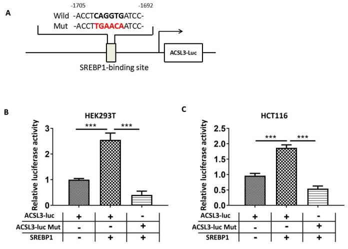
A
-1705
-1692
Wild -ACCTCAGGTGATCC-
Mut -ACCTTGAACAATCC-
ACSL3-Luc
SREBP1-binding site
B
HEK293T
***
***
Relative luciferase activity
C
HCT116
***
***
Relative luciferase activity
ACSL3-luc + + -
ACSL3-luc Mut - - +
SREBP1 - + +
